# Smoking increases risk of complication after musculoskeletal surgery: analysis of single immune parameter to predict complication risk

**DOI:** 10.17179/excli2024-7306

**Published:** 2024-07-13

**Authors:** Leyla Tümen, Lena Pollmann-Schweckhorst, Regina Breinbauer, Mohammad M. Hammour, Romina H. Aspera-Werz, Gunnar Blumenstock, Tina Histing, Maximilian M. Menger, Sabrina Ehnert, Andreas K. Nüssler

**Affiliations:** 1 Department of Trauma and Reconstructive Surgery, Eberhard Karls University Tübingen, BG Trauma Center Tübingen, Siegfried Weller Institute for Trauma Research, 72076 Tübingen, Germany; 2 Department of Trauma and Reconstructive Surgery, Eberhard Karls University Tübingen, BG Trauma Center Tübingen, 72076 Tübingen, Germany; 3 Department of Medical Biometry, Eberhard Karls University Tübingen, 72076 Tübingen, Germany

**Keywords:** smoking, systemic immune inflammation index, SII, complications, surgery, trauma

## Abstract

Smoking is the most significant and modifiable risk factor for a range of conditions, including cancer, cardiovascular and respiratory diseases. Furthermore, it significantly reduces bone mass and increases the risk of fragility fractures due to its detrimental effects on bone metabolism and regeneration. Moreover, smoking is a known cause of chronic systemic inflammation, leading to an imbalance of cytokines. Comprehending the pathological mechanisms that underlie cytokine production and its impact on post-surgical healing is essential to prevent post-surgical complications. The present study recruited a total of 1144 patients, including 897 patients, among them non-smokers (*N = 413*), current smokers (*N = 201*) and ex-smokers (*N = 283*). Human proteome profiler arrays were used to screen for smoking-dependent differences in the serum cytokine and protein profiles, after matching samples for age, gender, body mass index (BMI), alcohol use, and diabetes risk. Cytokines and immune checkpoint proteins such as CD28, B7-1, MIG, TGFβ2 and IL-1α/β were quantified by ELISA. Our study demonstrates a comprehensive understanding of the relationship between smoking, the development of complications, the systemic immune inflammation index (SII) and cytokine/protein levels. We found that a comparison of non-smokers, former smokers, and active smokers in our study cohort did not exhibit significantly altered cytokine and protein serum levels although other studies reported differences between smokers and non-smokers. We were unable to identify single blood circulating markers that could predict complications in smokers after trauma. However, we found the ratio of women to men to be inverted between non-smokers and active smokers resulting in a ratio of 0.62 in smokers. Furthermore, we demonstrate a higher complication rate, longer hospitalizations and elevated SII values among smokers, indicating an involvement of the immune system.

See also the graphical abstract(Fig. 1).

## Introduction

Smoking is unequivocally the most significant and potentially modifiable risk factor, contributing not only to public health issues but also to an economic burden of over $ 193 billion in annual healthcare costs in the United States (Truntzer et al., 2015[92]; Scherubl 2021[75]; CDC, 2008[11]). According to a study conducted by the Robert Koch Institute (RKI) in 2022, approximately 55 % of adults in Germany are smokers, with the group being divided into former (26.7 %) and active smokers (28.9 %) (Starker et al., 2022[87]; RKI[70]). Furthermore, it is common knowledge that smokers have longer hospital stays than non-smokers (Rezaei et al., 2016[69]; Ehnert et al., 2019[24]; Abate et al., 2013[1]). Smoking represents a major risk factor for a plethora of diseases such as cancer, cardiovascular and respiratory conditions and reproductive disorders (Ehnert et al., 2019[24]). In addition, smoking also affects bone metabolism and regeneration, resulting in reduced bone mass and, accordingly, a higher rate of fragility fractures (Daniell, 1976[18]; Scolaro et al., 2014[78]). Moreover, there is strong evidence that smoking causes post-surgical complications including impaired wound and delayed fracture healing (Hess et al., 2020[33]; Mills et al., 2011[53]). These complications include a twofold increase in the risk of non-union and infections (Pearson et al., 2016[61]; Gaston and Simpson, 2007[27]). As a consequence, smokers experience extensive revision surgery, resulting in additional pain and loss of function in the affected limb. Due to the prolonged rehabilitation, smoking represents a critical strain on our healthcare system (Kanis et al., 2005[40]). In 2018, smoking incurred direct costs of approximately € 30 billion in Germany, with smoking-related medical costs alone amounting to € 27 billion (Effertz, 2019[23]).

In general, adults aged 65 and older are much less likely to smoke than younger age groups. As a result, smokers tend to be younger than ex-smokers (Starker et al., 2022[87]). The inflammatory response is a crucial prerequisite for the successful process of wound healing and regeneration (Loi et al., 2016[50]). Interestingly, there is contradicting information on the effects of smoking on the expression of inflammatory markers. Javed et al. (2015[39]) demonstrated that interleukin (IL)-1β and IL-6 levels are significantly higher in the salivary of smokers when compared to non-smokers. Moreover, Suzuki et al. (2016[88]) showed that smokers exhibit higher levels of immunoglobulin A (IgA) and tumor necrosis factor (TNF)-α in the saliva than non-smokers. In contrast, several other studies indicate that smoking inhibits toll-like receptor (TLR)-2 and-4-dependent expression of pro-inflammatory cytokines and compromizes immunity after bacterial infection (Chen et al., 2007[12]; Lugade et al., 2014[51]). This is supported by epidemiological studies indicating that smokers are prone to developing infections after injury (Knapik and Bedno, 2018[43]). However, there is no information on the cytokine and immune checkpoint protein profile of non-smokers and smokers after musculoskeletal surgery and how cytokine and protein levels may serve to predict the healing process.

Therefore, this study aimed at analyzing the blood circulating cytokine and immune checkpoint protein profile of smokers and non-smokers prior to a musculoskeletal surgery and its relation to the clinical outcome, in order to identify a potential marker predictive of complications in smoking patients, as not all smokers develop them. 

## Materials and Methods

### Ethics statement

The study was conducted in strict accordance with the Declaration of Helsinki (1964) and its latest amendment. Patients were interviewed and clinically relevant data were collected according to ethical vote 346/2015B02, amended on 30.03.2020 and accepted on 09.07.2020. Blood collection was done during a routine blood sampling after all participants gave their written informed consent.

### Patient recruitment and survey

Between July 2020 and October 2022, patients were recruited at a level 1 trauma center including the departments of traumatology, septic surgery and arthroplasty. Patients unwilling or unable (communication problems resulting from mental disability, language difficulties, or dementia) to participate, were excluded from the study. The screening process included bedside interviews and follow-up interviews after 3 months. Patient data, including smoking status and cigarette consumption, was documented. Complications were identified using the hospital's IT system after the patient's discharge. Complications were defined as death, infections, wound healing disorders, further necessary operations, and thrombosis. All complications were evaluated consistently during hospitalization and 3 months post-surgery. It is important to note that the evaluation process was thorough and consistent, ensuring accurate identification of any complications. Due to the fact that the Clavien-Dindo classification is based on the therapy required to treat a complication, we have developed our own post-surgical classification system (Table 1(Tab. 1)), to identify complications beyond those identified by the Clavien-Dindo classification (Clavien et al., 2009[16]). 

### Blood sampling

Approximately 10 mL of blood (5 mL for serum and 5 mL for EDTA plasma) was collected during preoperative routine blood sampling. The samples were centrifuged at 1000 g at 4 °C for 10 minutes within 60 minutes of collection. Throughout this process, the samples were maintained on ice. The resulting serum and plasma samples were stored as aliquots at -80 °C until further use. In addition, the systemic immune inflammation index (SII) was calculated using the following equation: SII = P x N/L, with P, N, and L referring to platelet, neutrophil, and lymphocyte counts, respectively (Hu et al., 2014[36]).

### Human proteome profiler arrays

Relative serum levels of cytokines and proteins were determined with the Human Cytokine Array C5 and Human Immune Checkpoint Array C1 (RayBiotech^®^ - BioCat, Heidelberg, Germany). Arrays were performed following the manufacturer's instructions, with sample incubation at +4 °C overnight. Chemiluminescent signals were recorded using a charge-coupled device camera (INTAS, Göttingen, Germany). The signal intensities were quantified with the ImageJ software (version 1.54f, NIH, Bethesda, MA, USA). Signal intensities were normalized to the six positive controls provided on each array membrane. To compensate for differences in signal intensity between the different target proteins, a z-score normalization was performed using the following equation x' = (x - μ) / σ, with μ = mean and σ = standard deviation of all samples for the respective target protein.

### Enzyme-Linked Immunosorbent Assay (ELISA)

MCP-1, Eotaxin 3, CD28, B7-1, B7-2, CTLA-4, MIG, IL-1α, IL-1β, IL-13, PDGF-BB, TGFβ2 and TIMP-1 were quantified in the serum samples using commercially available ELISA kits (Table 2(Tab. 2); References in Table 2: Chen and Flies, 2013[14]; Defrance et al., 1994[20]; Deshmane et al., 2009[21]; Murphy and Weavers, 2018[55]; Pardoll, 2012[59]; Petkovic et al., 2004[62]; Phipps et al., 2012[64]; Rudd et al., 2009[73]; Schoeps et al., 2021[77]; Tokunaga et al., 2018[91]; Van Coillie et al., 2020[93]; Wang et al., 2023[96]), following the manufacturer's instructions. These 13 cytokines and proteins were selected as previous studies have either shown a change in their levels following smoking or they have been shown to have an effect on bone metabolism and remodeling (Wang et al., 2022[94]; Ehnert et al., 2019[24]; Haytural et al., 2015[32]; Miyamoto et al., 2009[54]; Onoe et al., 1996[58]; Axmann et al., 2008[4]; Ahmadi et al., 2020[3]; Xie et al., 2014[103]). 

### Statistics

Statistical analysis was performed using GraphPad Prism (version 9.3, El Camino Real, California, CA, USA) and JMP (version 17.2.0, SAS Institute Inc., Cary, NC, USA). Results are presented as boxplots, bar charts (mean ± standard error of the mean (SEM)), scatter plots (median with 95 % confidence interval (CI)) or heat maps (z-score). The heat map was performed using the website https://software.broadinstitute.org/morpheus/. The number of donors (N) is clearly indicated in the figure legends. All serum analyses were performed in technical duplicates (n = 2). Data comparison between the two groups was performed with Student's t-test if the normal distribution was apparent, otherwise Mann-Whitney U test was used. Categorical data were analyzed using chi-squared tests.

For multiple groups, ordinary one-way ANOVA followed by Tukey's multiple comparison test was performed when normally distributed. Otherwise, the Kruskal-Wallis test was used. A significance level of *p *< 0.05 with α = 0.05 was set as a threshold to determine statistically significant results for all experiments.

Receiver operating characteristics (ROC) were calculated to evaluate the diagnostic performance, involving the calculation of the area under the curve (AUC), 95 % CI, sensitivity, specificity and the Youden index for diagnostic accuracy.

## Results

### Patient recruitment and description of the study cohort

**Figure 2:**
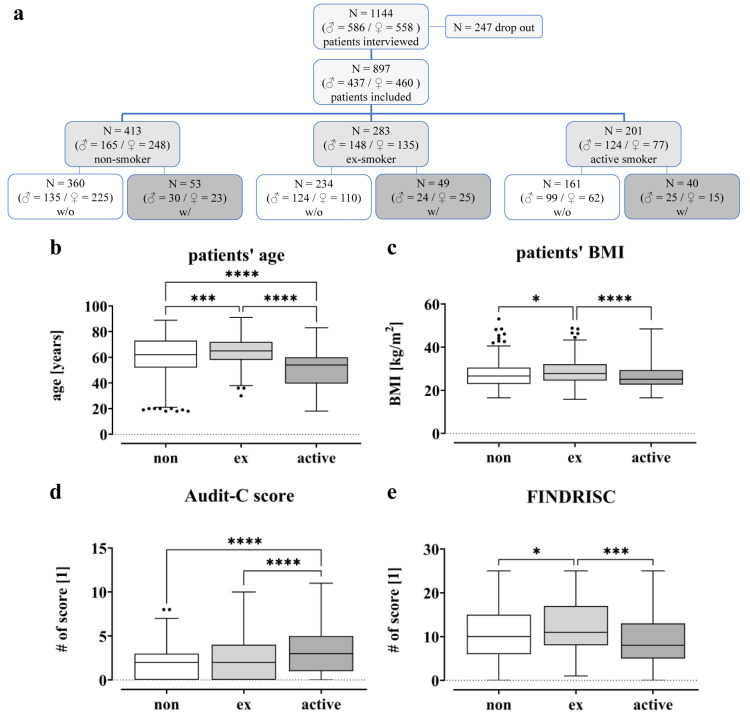
Overview of the study population. (a) CONSORT Flow Diagram: A total of 1144 patients were interviewed for this study during their hospital stay in the trauma surgery department between July 2020 and October 2022. Due to pre-existing/diagnosis- or procedure-related complications, 247 patients were excluded from the study. Based on their smoking behavior, they were divided into three different groups. (b) Patients' age is given in years. (c) Patients' BMI is given in kg/m^2^. (d) Alcohol Use Disorders Identification Test (AUDIT-C). (e) Finnish Diabetes Risk Score (FINDRISC). Data displayed as boxplots (Box and Whiskers-Tukey to visualize outliers), significant differences were determined by one‐way analysis of variance (ANOVA) with a p-value of less than 0.05 considered as statistically significant (* p < 0.05, ** p < 0.01, *** p < 0.001 and **** p < 0.0001).

Between July 2020 and October 2022, a total of 1144 patients (586 men and 558 women), hospitalized at the level 1 trauma center due to trauma or upcoming elective surgery, were randomly included in this study. Of these, 247 patients were excluded due to pre-existing/diagnosis- or intervention-associated complications. In total, 897 patients were included in the study. 361 of these patients underwent joint arthroplasty, 110 were from geriatric traumatology, 78 patients from septic surgery and 348 from trauma and spine surgery. Based on their smoking behavior, patients were divided into different groups, *i.e.* non-smokers with 0 PY (*N = 413*), ex-smokers (*N = 283*) and active smokers (*N = 201*) (Figure 2(Fig. 2)). In addition, a distinction was made between patients with complications (w/) and controls (w/o). Among the non-smokers (413 total), 53 patients developed a complication after surgery, while 49 patients in the ex-smoker group (283 in total) suffered a complication. In the group of active smokers (201 in total) complications occurred in 40 patients. 

For the study cohort, age, BMI, Alcohol Use Disorder Identification Test score (AUDIT-C) and Finnish Diabetes Risk Score (FINDRISC) were examined in detail in relation to cigarette consumption (Figure 2b-e(Fig. 2)). The FINDRISC was used to assess the risk of developing diabetes, while the AUDIT-C was performed to identify alcohol use disorders.

Figure 2b(Fig. 2) shows that active smokers are younger (50.3 ± 1.1) than ex-smokers (64.4 ± 0.6; p < 0.0001) and non-smokers (60.0 ± 0.8; p < 0.0001), with the ex-smokers having the highest average age. Additionally, the active smokers appear to have lower BMI levels (26.4 ± 0.4) compared to non- (27.4 ± 0.3) and former smokers (28.6 ± 0.3), with all groups having high BMI levels overall (Figure 2c(Fig. 2)). The data also show that active smokers have higher AUDIT-C values (3.1 ± 0.2) compared to former (2.3 ± 0.1; p < 0.0001) and non-smokers (2.0 ± 0.1; p < 0.0001; Figure 2d(Fig. 2)). In contrast, FINDRISC values were highest in former smokers (12.9 ± 0.4) and lowest in active smokers (10.2 ± 0.5; Figure 2e(Fig. 2)).

### Higher rates of complications and longer hospital stays in active smokers

**Figure 3:**
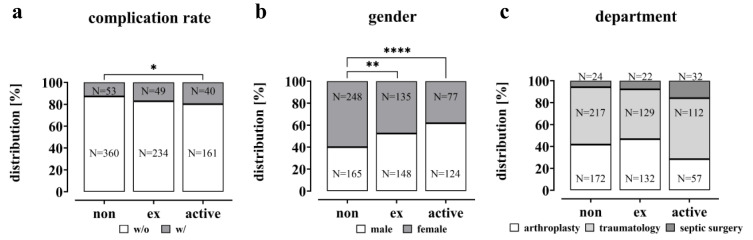
Complication rate, gender and departmental distribution in the study cohort visualized as a bar plot. (a) The complication rate is given in % and the total number (N), significance was determined by Chi-square test. (b) Gender distribution within the groups is given in % and total numbers (N), significant differences were determined by Chi-square test with a p-value of less than 0.05 considered as statistically significant (* p < 0.05, ** p < 0.01, *** p < 0.001 and **** p < 0.0001). (c) Departmental distribution within the groups is given in % and total numbers (N).

The study investigated the impact of smoking on the clinical outcomes of the patient cohort. The complication rate was significantly increased in active smokers compared to non-smokers (non-smokers 12.8 %, active smokers 19.9 %; p = 0.0219; Figure 3a(Fig. 3)). However, the rate was not significantly higher among former smokers (17.3 %) compared to non-smokers. The data also show that smoking was more prevalent among male patients than female patients (61.7 % male active smokers vs. 40.0 % male non-smokers; p < 0.0001 and 52.3 % male ex-smokers, p = 0.0013, Figure 3b(Fig. 3)). Within the study population, it was found that smokers had a lower rate of planned endoprosthetic procedures compared to ex- and non-smokers. Additionally, smokers had a higher incidence of traumatic and especially infectious events (Figure 3c(Fig. 3)).

**Figure 4:**
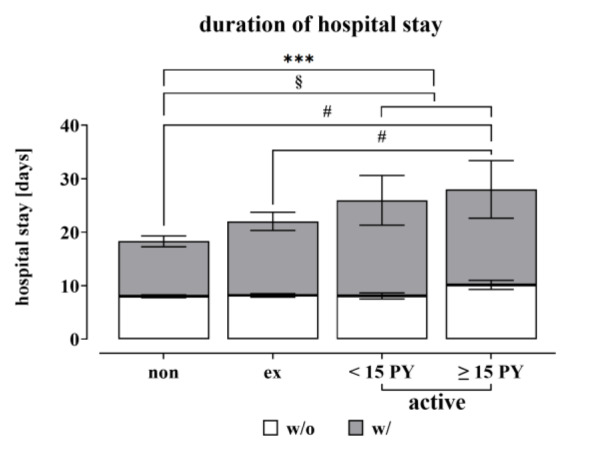
Duration of hospitalization of the study population based on smoking behavior. For the analysis, a distinction was made between non-smokers with 0 pack-years (N=413), ex-smokers (≥ 1-year ex-smoker regardless of PY, N=283) and active smokers (N=201). The active smokers were divided into moderate (< 15 pack-years, N=96) and heavy smokers (≥ 15 pack-years, N=105). Data are summarized as mean ± SEM; significant differences were determined by one‐way analysis of variance (ANOVA) with a p-value of less than 0.05 considered as statistically significant. “*” indicates the significance of the entire group, while “#” denotes the significance of the population without complication and “§” shows the significant differences of the group with complication (*, #, § p < 0.05, **, ##, §§ p < 0.01, ***, ###, §§§ p < 0.001 and ****, ####, §§§§ p < 0.0001).

Overall, 142 patients across all groups experienced complications, such as infection, wound healing disorders, required revision surgery, thrombosis, etc. within three months after surgery. As anticipated, patients who experienced complications had a significantly longer hospital stay compared to those who did not develop any adverse events. When comparing hospitalizations, it is evident that active smokers (10.9 ± 0.8 days) experienced a significantly longer hospitalization period than non-smokers (8.3 ± 0.3; p = 0.0004) (Figure 4(Fig. 4)). As the longer hospital stay of active smokers may be due to the higher complication rate, the hospital stays of the groups with and without complications were examined. 

From Figure 4(Fig. 4) it can be concluded, that active smokers with complications have a longer stay in hospital than non-smokers with complications.

Looking more closely at the time of hospitalization in the group without complications and dividing the active smokers into the subgroups, heavy smokers with ≥15 pack-years (PY) in particular have a longer hospital stay (10.2 ± 0.9, p = 0,0167) and this is also significant in comparison with ex-smokers (8.2 ± 0.3; p = 0,0499) and non-smokers.

### High Systemic Immune-Inflammation Index in smokers

**Figure 5:**
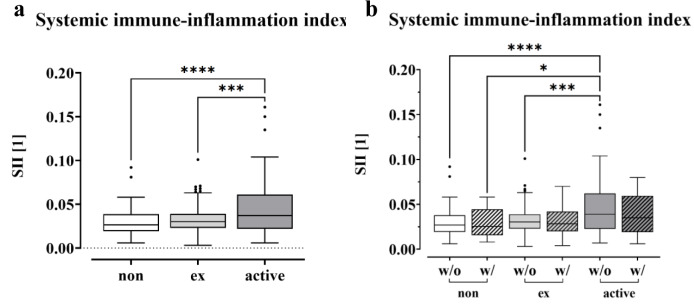
Correlations between smoking behavior and SII. The Systemic Immune-Inflammation Index (SII) of non-smokers (0 PY), ex-smokers and active smokers is displayed as a boxplot (Box and Whiskers-Tukey). The SII was calculated using the following equation, SII = P x N/L, blood cell counts of P platelets, N neutrophil and L lymphocytes, significance was determined through one-way analysis of variance (ANOVA) with a p-value less than 0.05 being considered statistically significant (*p < 0.05, **p < 0.01, ***p < 0.001, and ****p < 0.0001) as the data followed a normal distribution. (a) Comparison of the SII levels (with and without complication) between non-, ex- and active smokers. (b) SII values for non-, ex- and active smokers, distinguishing between complications and no complications.

To gain insight into potential mechanisms of immunosuppression in smokers, we calculated the systemic immune inflammation index (SII) for non-smokers (0 PY), ex-smokers and active smokers using the following equation: SII = P x N/L, with P, N, and L referring to platelet, neutrophil, and lymphocyte counts, respectively (Hu et al., 2014[36]). As shown in Figure 5a(Fig. 5), the SII was significantly increased in smokers (p < 0.0001) compared to non-smokers at 0 PY, which was also the case for former smokers (p = 0.0001). When comparing the SII values and distinguishing between patients who develop complications and those without, we see a trend of decrease for both ex-smokers and active smokers when complications occur (Figure 5b(Fig. 5)). The highest values were observed in active smokers without complications, which were significantly higher compared to all groups except for active smokers with complications. When comparing the levels within the group of non-smokers, former and current smokers, a slight decrease can be seen in the group with complications. However, no significant differences were found.

### Altered cytokine and protein levels in smokers

**Figure 6:**
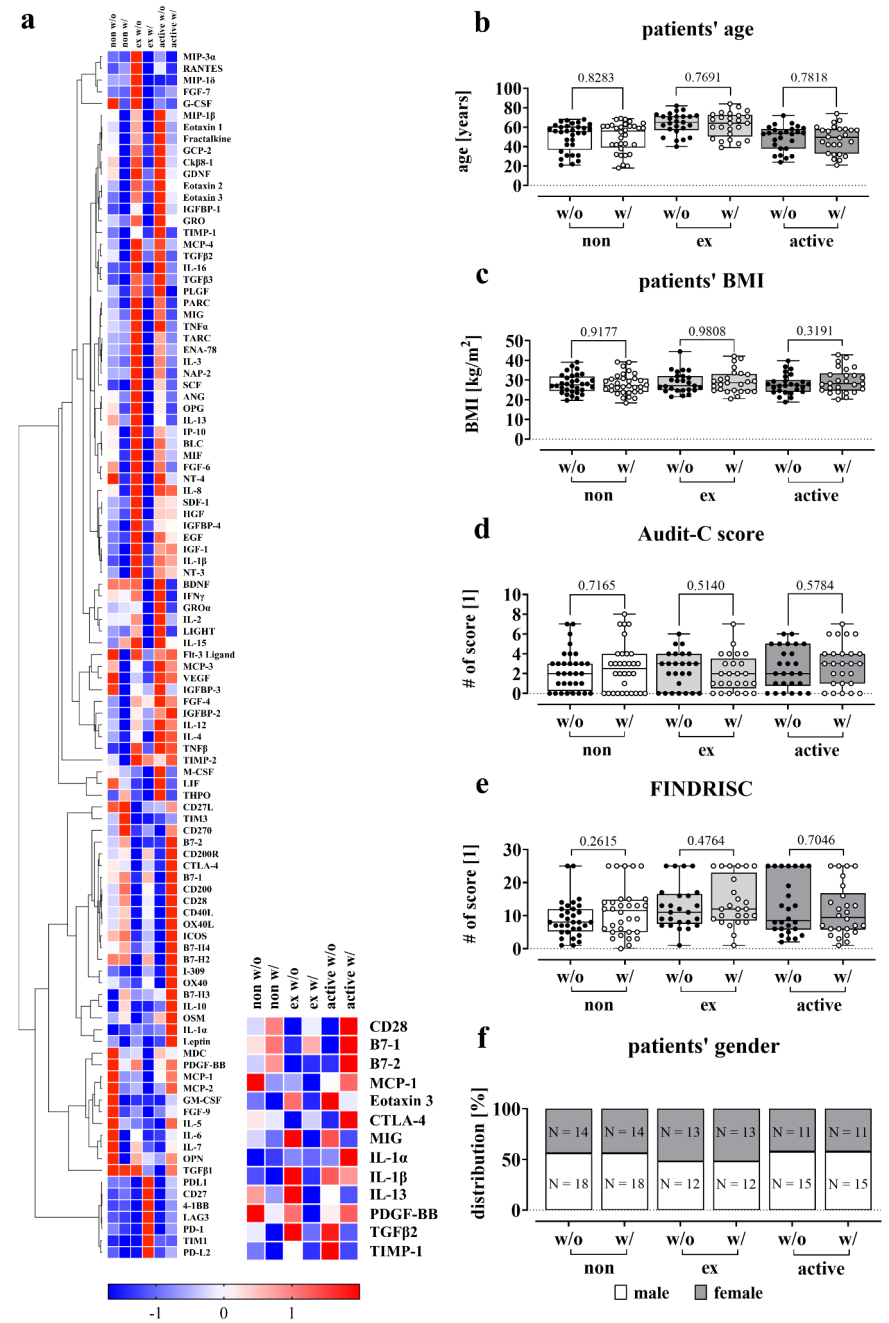
Effect of smoking on circulating factors in the blood. (a) Relative cytokine and immune checkpoint protein levels in serum samples from non-smokers (0 PY), ex-smokers and active smokers were determined using the RayBiotech® Human Cytokine Array C5 and Human Immune Checkpoint Array C1 (0 PY: N = 32, ex: N = 25, active: N = 26). For the heatmap, signal intensities were normalized using z-score by following the equation x' = (x - μ) / σ, defining μ as the mean and σ as the standard deviation of the samples. Underrepresented cytokines and proteins are colored blue, overrepresented cytokines/proteins are colored red. (b-f) Description of the study population for the C5 and C1 Array measurements based on smoking behavior with analyses of age, BMI, Audit-C score, FINDRISC and gender. Data shown as boxplot (Box and Whiskers) or bar plot, significance was determined by Student's t-test. (b) Patients' age is given in years. (c) Patients' BMI is given in kg/m^2^. (d) Audit-C score (Alcohol Use Disorders Identification Test) for the detection of patients with hazardous alcohol consumption or active alcohol use disorders. (e) FINDRISC (Finnish Diabetes Risk Score) to identify patients at high risk for type 2 diabetes. (f) Gender distribution within the groups, given in % and total numbers (N). P-values are given at the top of the brackets.

To gain insight into possible regulatory mechanisms, we analyzed cytokine and protein levels in the blood of patients with and without complications. To eliminate potential confounding factors, we formed homogeneous groups with similar values for age, BMI, Audit-C and FINDRISC. We ensured that the subgroups were comparable in terms of these variables to reduce the likelihood of any confounding effects (Figure 6b-f(Fig. 6)). The screening therefore included 32 non-smokers per group (with and without complication), 25 ex-smokers and 26 active smokers. 

Cytokine and protein levels were measured using the RayBiotech® Human Cytokine Array C5 and Immune Checkpoint Array C1. As shown in Figure 6a(Fig. 6), blood serum levels of cytokines and proteins differed in various ways between ex-smokers and active smokers. The serum levels of T-cell activation and regulation markers, specifically CD28, B7-1, and B7-2, were lower in both ex-smokers and active smokers in the absence of complications. However, with complications, the levels increased, particularly in active smokers. Notably, there was no significant difference observed between ex-smokers with and without complications.

Regarding interleukin 1α and 1β, there is a partly contradictory picture, although both cytokines activate T-cells as well as macrophages (Murphy and Weaver, 2018[55]). IL-1β is particularly elevated in former and active smokers in the absence of complications, however lower levels are found when complications occur, which is not pronounced for the group of active smokers. For IL-1α, there was an isolated elevation in the complication group of smokers.

CTLA-4 which plays an important role in maintaining self-tolerance by inhibiting excessive T-cell activation is slightly lower in non-smokers with complications than in the group without (Van Coillie et al., 2020[93]). In contrast, ex- and active smokers without complications have lower levels of CTLA-4, which is particularly higher in active smokers with complications.

MCP-1, Eotaxin 3 and TIMP-1, all involved in the chemotaxis of immune cells, are regulated in different ways (Deshmane et al., 2009[21]; Schoeps et al., 2021[77]; Petkovic et al., 2004[62]). MCP-1 is reduced in people who do not currently smoke, i.e. non-smokers and ex-smokers, when complications occur, whereas the opposite is the case in active smokers. 

The levels of TIMP-1 were lower for non-smokers and ex-smokers but increased for active smokers without complications. Eotaxin 3 however was particularly elevated in former and current smokers in the control group.

Similar findings were observed for MIG and TGFβ2, which are involved in immune cell proliferation or differentiation (Tokunaga et al., 2018[91]; Wang et al., 2023[96]). In contrast, PDGF-BB, which is responsible for the migration of mesenchymal stem cells, showed a decrease in the group of complications for non- and ex-smokers (Phipps et al., 2012[64]). However, in active smokers with complications, there was an increase observed.

**Figure 7:**
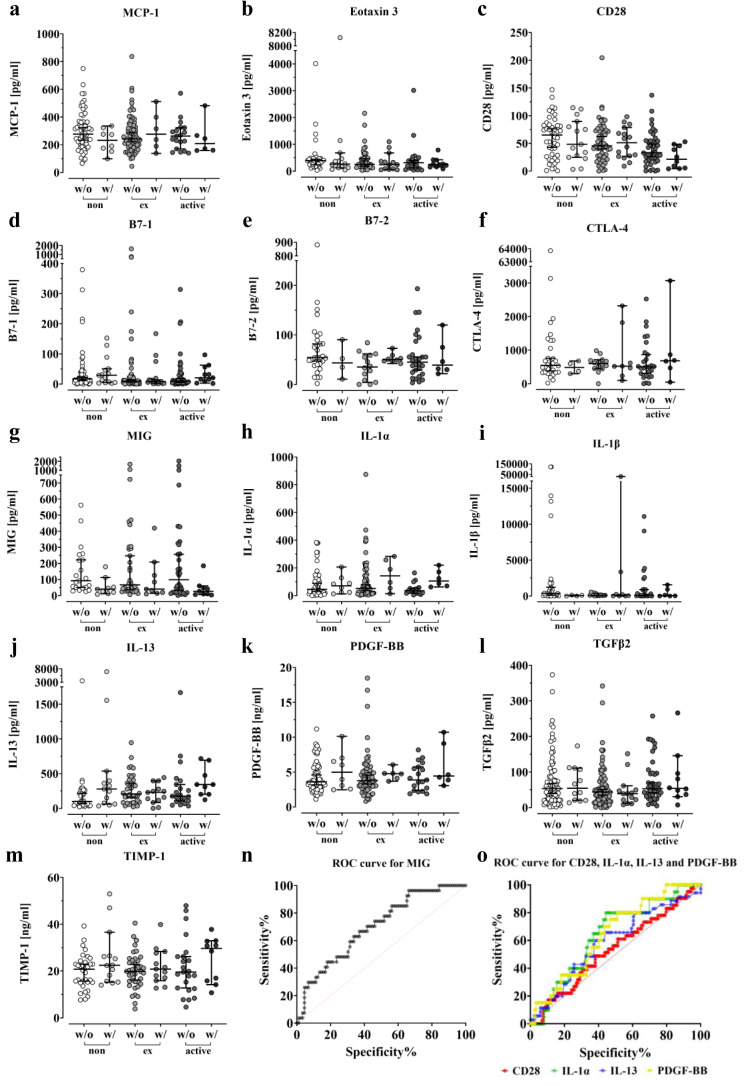
Impact of smoking on serum levels of several proteins. Differentiation was made in the analysis between non-smokers, ex-smokers and active smokers. All ELISAs were performed in duplicate. Error bars indicate the median ± 95 % confidence interval and significance was determined by Kruskal-Wallis H-test with a p-value of less than 0.05 considered statistically. The serum levels of the MCP-1 (a), Eotaxin 3 (b), CD28 (c), B7-1 (d), B7-2 (e), CTLA-4 (f), MIG (g), IL-1α (h), IL-1β (i), IL-13 (j), PDGF-BB (k), TGFβ2 (l) and TIMP-1 (m) were measured in both the complication and control groups. (n) Receiver operating characteristics (ROC) curve for MIG (AUC = 0.70), (o) Receiver operating characteristics (ROC) curve for CD28 (AUC = 0.53), IL-1α (AUC = 0.64), IL-13(AUC = 0.59) and PDGF-BB (AUC = 0.64).

After screening for more than 100 different cytokines and proteins with the arrays, we decided to measure the preoperative blood serum levels of 13 different markers summarized in Figure 7(Fig. 7) by ELISA in order to confirm the observed differences in the arrays. 

For patients with infection, blood samples could not be obtained prior to the initial emergency procedure. Hence, we excluded these patients from the analysis, since the protein levels could be elevated due to the prior surgery or the inflectional event. Figure 7c(Fig. 7) and Figure 7e(Fig. 7) illustrate a decrease in serum levels of CD28 and B7-2 in former and active smokers, both essential for T cell activation and survival (Chen and Flies, 2013[14]). In smokers, the levels of CD28 and B7-2, which are already reduced, decreased further compared to non-smokers when complications occurred.

The levels of IL-1α remained relatively constant in the respective control groups regardless of smoking status and increased when complications occurred (Figure 7h(Fig. 7)). IL-13 levels increased in smokers, particularly former smokers, when compared to non-smokers, and also increased in the complication group, particularly in non-smokers and active smokers (Figure 7j(Fig. 7)). Similar results were seen with PDGF-BB (Figure 7k(Fig. 7)). Both ex-smokers and active smokers had slightly higher basal levels compared to non-smokers, which increased further when complications occurred. However, the increase in ex-smokers and active smokers was not as pronounced as in non-smokers. Looking at Figure 7g(Fig. 7), it can be seen that patients who smoke have higher median MIG levels compared to non-smokers. In patients with a history of smoking, the elevated MIG levels decrease and fall below those of non-smokers at 0PY. However, upon closer inspection, it becomes apparent that MIG levels gradually decrease with the onset of complications, regardless of smoking status.

To determine the diagnostic accuracy for several markers, ROC curves were calculated.

The AUC, area under the curve, values for CD28, IL-1α, IL-13, and PDGF-BB were 0.53, 0.64, 0.59, and 0.64, respectively (Figure 7o(Fig. 7)). MIG had the highest AUC at 0.70 (Figure 7n(Fig. 7)). With a calculated threshold of < 213.9 pg/ml, MIG showed a sensitivity of 0.963 and a specificity of 0.337 for detecting a complication, resulting in a Youden index of 0.30.

## Discussion

Smoking can cause inflammatory and autoimmune diseases through various mechanisms, including genetic changes, increased oxidative stress and free radicals (Wang et al., 2022[94]). Furthermore, smoking is in general a well-established risk factor for osteoporosis, fragility fractures, and related post-surgical complications such as infections or delayed wound healing (Mills et al., 2011[53]; Hess et al., 2020[33]; Rudang et al., 2012[72]; Sloan et al., 2010[84]; Scolaro et al., 2014[78]; Abate et al., 2013[1]; Gronkjaer et al., 2014[29]; Sorensen, 2012[85]; Knapik and Bedno, 2018[43]; Porter and Hanley, 2001[66]). The literature contains numerous theories on the impact of smoking on fracture healing including reduced blood supply, increased levels of reactive oxygen species, lack of antioxidants and vitamins, and toxic effects on osteoblasts/osteoclasts and calcitonin (Sloan et al., 2010[84]; Gaston and Simpson, 2007[27]; Abate et al., 2013[1]). 

Moreover, smoking has been proven to prolong the healing process of fractures and wounds (Abidi et al., 1998[2]; Schmitz et al., 1999[76]) and it is well established that both, direct and indirect exposure to smoke has a detrimental impact on bone mineral content (Abate et al., 2013[1]). It is suggested that smoking reduces bone strength by increasing the size of the diaphyseal medullary cavity and decreasing the size of the epiphyseal trabecular bone, although the exact cause remains unknown (Wust et al., 2010[102]). Smoking notoriously impairs wound healing (Mills et al., 2011[53]), but the mechanisms behind this phenomenon are not yet understood. 

As demonstrated in this study, there is a correlation between smoking and postoperative complications. Such occurrences are also significantly more prevalent among individuals with a low socioeconomic status. Furthermore, an elevated risk of morbidity and mortality is observed (Bartley 1994[7]; de Jager et al., 2022[19]; Mehaffey et al., 2020[52]; Reames et al., 2014[68]; Bennett et al., 2010[8]). Nevertheless, a 2012 study demonstrated that there was no association between educational status and mortality when income was taken into consideration and that lower income was positively associated with mortality after adjustment for education (Sabanayagam and Shankar 2012[74]). However, a review of the literature did not identify any evidence of an increase in inflammatory markers. 

Obesity, which is associated with a low socioeconomic status (de Jager et al., 2022[19]; Bartley, 1994[7]; Bambra and Eikemo, 2009[5]) is associated with an increase in several inflammatory markers, leading to chronic low-grade inflammation. Adipose tissue in obese has been shown to secrete pro-inflammatory markers due to a shift in immune cells from anti-inflammatory M2 macrophages (producing IL-10, IL-5 and interferon-γ) to pro-inflammatory M1 macrophages (TNF-α, IL-17, IL-6 and IL-1β). This leads to altered serum levels of the aforementioned inflammatory cytokines, which have been shown to exert significant effects on diabetes, hypertension, and other chronic conditions. The increasing inflammatory reaction in obesity can be explained at least in part as follows: the enlargement of fat cells leads to an increase in the distance to the nourishing vessels, creating hypoxic conditions. This could lead to fibrosis of the adipose tissue and the resulting infiltration of macrophages, which trigger the local inflammatory reaction. Furthermore, adipose tissue can secrete approximately 50 different hormones, affecting the immune system and metabolism (Khanna et al., 2022[41]; Ellulu et al., 2017[25]). 

Although several factors have been shown in the literature to be associated with an increased risk of complications, we decided to focus on smoking, since our previous study cohort of 817 participants confirmed that smoking is a major risk factor for complications like infection, wound healing, need for revision surgery, thrombosis, and/or death. Compared with participants with risk for malnutrition or daily alcohol intake, the incidence of complications after total joint arthroplasty was higher in former and active smokers (Ehnert et al., 2019[24]).

For the healing process to occur, growth factors and cytokines need to be secreted for instance by fibroblasts migrating into the wound area (Barrientos et al., 2008[6]; Werner and Grose, 2003[98]; Wang et al., 2024[95]). However, smoking inhibits the biological function of fibroblasts directly (Wong and Martins-Green 2004[100]; Sloan et al., 2010[84]; Nakamura et al., 1995[56]). The importance of these cells is clearly demonstrated by a study conducted by Oe et al. (2007[57]), in which they isolated fibroblast-like progenitor cells from the hematoma of tibial fractures, which could be differentiated *in vitro* into osteocytes, chondrocytes and adipocytes. These findings underscore the critical role of these cells in the regeneration of bone tissue.

Other studies have shown that nicotine directly alters a number of cytokine genes associated with neovascularization and osteoblast differentiation, such as vascular endothelial growth factor (VEGF), bone morphogenetic protein (BMP)-2, -4 and -6, basic fibroblast growth factor (bFGF) and collagen I/II (Theiss et al., 2000[90]). Smoking thus disrupts the homeostasis of the immune system by modulating immunoregulatory activities leading to inflammation (Wang et al., 2022[94]), which is controlled by a complex network of secondary messengers, such as cytokines and chemokines, being part of both innate and adaptive immunity (Wong and Fish, 2003[101]; Wang et al., 2022[94]; Commins et al., 2010[17]).

However, the precise mechanism by which smoking affects inflammation remains unclear. Thus, we examined the impact of smoking on cytokine and protein levels prior to musculoskeletal surgery to identify a blood parameter that can be measured in a routine laboratory and accurately predict complications. 

The present study involved 897 patients with various musculoskeletal diseases who had to undergo surgery and were categorized into three groups, based on their smoking status: non-smokers, ex-smokers, and active smokers. The data were then analyzed, including the rate of complications, gender distribution, length of hospital stay and the score of the systemic immune inflammatory response. Moreover, the levels of various cytokines and proteins in the patients' serum were measured *via* arrays and more specified by ELISAs.

We were able to show, that over 50 % of the patients who attend our clinic are smokers, either former smokers (31.6 %) or current smokers (22.4 %), which is in accordance with the 55 % smoking rate reported by the Robert Koch Institute (RKI) (Starker et al., 2022[87]; RKI[70]). Moreover, our study confirms the link between smoking and a higher rate of complications, consistent with the existing literature (Scolaro et al., 2014[78]). Due to the higher complication rate, the hospital stay was longer compared to non-smokers and former smokers, which is also supported by previous research (Abate et al., 2013[1]). 

Upon closer examination of the hospital stay, it is evident that active smokers experience longer hospitalizations than non-smokers and former smokers, even in the absence of complications, which suggests a delayed post-surgical recovery. This is the case even though smokers are, on average, younger than ex-smokers and have a lower BMI and FINDRISC, as demonstrated by our data. However, the hospitalization of ex-smokers is also prolonged to some extent, which is likely to be due to their older age, higher BMI and FINDRISC compared with the rest of the cohort. Nevertheless, with shorter hospital stays and lower rates of complications, former smokers seem to have a better recovery than young and active smokers. 

A more detailed look at the active smokers in our study cohort shows, that the proportion of men in this subgroup is higher than that of women. The calculated ratio of women to men, approximately 0.62, is consistent with the approximately 0.6 ratio reported by the World Health Organization for smokers in Germany (Hitchman and Fong, 2011[34]). Moreover, smokers are more prone to be hospitalized for traumatological and infectious events than non-smokers, which indicates both a compromized immune system and poorer bone quality. In contrast, a significant number of non-smokers were hospitalized for elective surgeries, such as total joint arthroplasty.

Since not all smokers develop a complication, we then measured cytokine and protein levels in the patients' blood to identify a potential predictive factor. 

The RayBiotech® Human Cytokine and Immune Checkpoint Array were performed to provide an overview of possible alterations and discrepancies in cytokine or protein levels between non-, ex- and active smokers. The control and complication groups were matched for gender, BMI, alcohol consumption, diabetes risk and age in order to ensure that any observed differences were not attributable to these factors. Subsequently, some of these molecules were quantified using ELISA. A preliminary literature search was therefore conducted with the objective of identifying existing findings on the molecules analyzed here. Following the identification of 13 different molecules in the literature, such as CD28, B7-1, CTLA-4, MIG, TGFβ2 and IL-1α/β, either shown to alter in smokers or affecting bone metabolism and remodeling, the study proceeded to quantify these molecules by ELISA (Wang et al., 2022[94]; Ehnert et al., 2019[24]; Haytural et al., 2015[32]; Miyamoto et al., 2009[54]; Onoe et al., 1996[58]; Axmann et al., 2008[4]; Ahmadi et al., 2020[3]; Xie et al., 2014[103]). 

None of these cytokines have been investigated in relation to smoking status and the development of postsurgical complications. Consequently, this study was undertaken in order to address this issue and proceeded to quantify the aforementioned molecules in serum by ELISA.

The use of serum was deemed appropriate, given that studies on the effects of sample treatment on the stability of cytokines had demonstrated that serum and plasma samples yielded comparable results, particularly when frozen without delay following collection (Flower et al., 2000[26]; Skogstrand et al., 2008[82]). As part of the study, Skogstrand et al. were able to demonstrate that prolonged storage of blood before separation into plasma or serum leads to an increase in the measurable concentration of several inflammatory markers, depending on the storage time and temperature. Therefore, a treatment at 4 °C and isolation of plasma and serum immediately after sample collection is recommended (Skogstrand et al., 2008[82]). It is important to note that the procedure for processing the blood samples in this case was highly standardized, with the samples being handled within the first hour of collection, during which time they were stored on ice. In contrast, other studies have demonstrated that the concentrations of cytokines in plasma differ from those in serum, which was attributed to the blood-clotting processes, given that a number of cytokines are released during this process (Guo et al., 2013[31]; Leng et al., 2008[46]). Other researchers have proposed that these discrepancies between plasma and serum concentrations can be explained by the plasma processing, whereby proteins such as fibrinogen and others are removed. This process also results in the removal of circulating proteins, such as cytokines, which frequently bind to these blood components (Leng et al., 2008[46]). Furthermore, the thawing of frozen samples was observed to result in a notable alteration in the concentrations of cytokines, while serum appeared to be less susceptible to this phenomenon compared to plasma (Parkitny et al., 2013[60]). Due to the aforementioned and in light of the previous findings conducted by our research group indicating a correlation between smoking and reduced levels of pro-inflammatory cytokines in serum, we elected to utilize the same test material in this investigation (Ehnert et al., 2019[24]).

CD28, B7-1 and B7-2 play a crucial role in the activation and differentiation of T-cells as essential costimulatory molecules (Chen and Flies, 2013[14]; Slavik et al., 1999[83]) and it is widely known that bone cells are under the influence of immune cells, particularly T-lymphocytes (Weitzmann and Pacifici, 2005[97]). Active T-cells, for example, can be destructive to bone and are a major source of RANKL (receptor activator of NF‐κB ligand) (Grassi et al., 2011[28]). Furthermore, Grassi et al. (2011[28]) defined osteoclasts as immunocompetent cells that attract and retain T-cells on the bone surface. B7-1, for instance, is significantly upregulated during osteoclast maturation, showing a fivefold increase, similar to APC (antigen-presenting cells) (Grassi et al., 2011[28]). 

In contrast, CTLA-4 (cytotoxic T lymphocyte antigen 4) is a co-inhibitory receptor that regulates the surface expression of B7-1 and B7-2 (Chen and Flies, 2013[14]; Qureshi et al., 2011[67]). CTLA-4 downregulates both B7-1 and B7-2 through trans-endocytosis (Qureshi et al., 2011[67]) and it promotes the activity of regulatory T-cells by binding to B7-1 and B7-2 and inhibits conventional T-cells (Chen and Flies 2013[14]; Rowshanravan et al., 2018[71]). Previous studies have shown increased levels of CTLA-4 have been found in patients with chronic obstructive pulmonary disease (COPD), a condition commonly associated with tobacco smoking (Shen et al., 2013[79]; Christenson et al., 2022[15]). Our study demonstrated that non-smokers with 0 pack-years and former smokers exhibit a reduced concentration of CTLA-4 when complications arise, compared to the corresponding control group. In contrast, smokers showed an increase in CTLA-4 levels, indicating impairment of the immune system in smokers (Zimmermann et al., 1997[109]; Simone et al., 2014[81]). For CD28, B7-1, and B7-2, active smokers showed slightly lower levels compared to non-smokers. Also, no significant difference was detected in patients with complications. 

Furthermore, we measured serum levels of MIG (monokine induced by interferon-γ (IFN-γ). In 2022, a study investigating the effects of smoking on the level of inflammation-related cytokines, found a significant increase in MIG, in human serum in both active and former smokers (Wang et al., 2022[94]). 

In the current study, we repeated this experiment and analyzed MIG levels in relation to complications. Unlike Wang et al. (2022[94]) we clearly demonstrated that smoking cessation leads to a decrease in serum levels of MIG. Also, MIG levels are lower in active smokers when complications occur compared to non-smokers with complications. 

It is important to note that MIG has been reported to have an osteoimmuno-modulating function (Phan et al., 2022[63]). Along with its receptor CXCR3, which is expressed on activated T-, B-cells and endothelial cells (Kwak et al., 2005[45]), it has been detected in osteoblasts and osteoclast precursors and has an inductive function on osteoclastogenesis leading to bone resorption (Phan et al., 2022[63]). It also impedes the proliferation, differentiation and mineralization of bone marrow stem cells (Huang et al., 2016[37]). Interestingly, MIG expression is directly induced by RANKL during osteoclastogenesis (Kwak et al., 2005[45]), which is an essential ligand for osteoclastogenesis (Yasuda, 2021[105]).

Our study and others have unequivocally demonstrated that smoking increases the proinflammatory response, as demonstrated by the rise in MIG levels (Wang et al., 2022[94]). Moreover, inflammation is associated with osteoclastic bone resorption (Kwak et al., 2005[45]), which implies that smokers with increased inflammation and higher serum MIG levels are at an elevated risk of fractures. Phan et al. (2022[63]) demonstrated this in a study by identifying an increased risk of hip fracture in men with high MIG levels, but not in women, though they would be expected to be at increased risk in the context of postmenopausal osteoporosis. However, comparing MIG levels between men and women, there were no differences within our cohort (data not shown).

Blocking MIG in the bone marrow with a neutralizing antibody prevented bone loss by inhibiting resorption and increasing bone formation (Liu et al., 2020[49]). Furthermore, it led to a significant reduction in CD4^+^ T-lymphocyte infiltration, which demonstrates the immunomodulatory effect of MIG in cardiac allograft vasculopathy (Yun et al., 2002[106]). Interestingly, scientists have shown that MIG interacts with vascular endothelial growth factor (VEGF), preventing it from binding to endothelial cells and osteoblasts, which in turn inhibits bone angiogenesis and osteogenesis (Huang et al., 2016[37]). Moreover, MIG appears to reduce the formation of type H blood vessels in bone, which is related to a loss of osteoprogenitor cells and a reduction in bone mass (Huang et al., 2016[37]; Liu et al., 2020[49]; Kusumbe et al., 2014[44]).

In the present study, we found a previously undescribed downregulation of MIG in the occurrence of complications compared to the respective control group. This was unexpected as the complication that has occurred is expected to lead to MIG-induced chemotaxis and hence recruitment of T-lymphocytes. Furthermore, our data suggest, that smoking cessation leads to a decrease of MIG. In order to assess the diagnostic potential of the measured proteins, receiver operating characteristics (ROC) were calculated. Notably, MIG exhibited the highest AUC of approximately 0.7. The analysis revealed a high sensitivity of 0.963, indicating a strong ability to accurately identify true positive cases. However, the specificity was only 0.337, suggesting a relatively high rate of false positive results. Due to this discrepancy, the analysis resulted in a Youden index of 0.3, indicating a low precise benefit of the diagnostic test. Similar situations were observed for the other proteins analyzed.

The implementation of a predictive marker could facilitate a more accurate estimation of the individual risk of postoperative complications, thereby enabling evidence-based clinical decision-making and the initiation of suitable preventive measures. In the most favorable circumstances, this could result in cost savings and the more effective utilization of resources.

In order for a marker like MIG to be considered a valid predictor, it must satisfy the following criteria: firstly, a correlation between the biomarker, the patient's smoking behavior and the occurrence of complications must be identified. Furthermore, in order to enhance the diagnostic accuracy of the test, the marker should be capable of exhibiting both high sensitivity and specificity. Finally, in order for the results of the test to be reliable, it must be guaranteed that there is no inconsistency in the results of repeated measurements conducted under different conditions and circumstances. It is therefore essential to ensure that other parameters have minimal impact on the marker. 

Provided the aforementioned criteria are met, it would be possible to predict complications, which would be of great clinical relevance. This could facilitate the adaptation of therapy, reducing the burden on hospitals and healthcare systems in terms of personnel and financial resources. Nevertheless, it is important to consider that the measurement of the marker must also be economically justifiable. Unfortunately, MIG was found to be unsuitable as a predictive indicator, exhibiting an extremely low level of specificity (0.337).

On closer inspection of the arrays and ELISA results for CD28, B7-2, MCP-1, IL-13 and TIMP-1, opposite results are apparent. This is due to the fact that the array groups were adjusted for age, BMI, FINDRISC and Audit-C, whereas no adjustment was made for the ELISA analysis. Thus, it is possible that age may influence blood serum levels of cytokines like IL-13, which has been described in a previous study (Li et al., 2014[48]). MCP-1, on the other hand, is known to be elevated in obesity and diabetes mellitus (Catalan et al., 2007[10]; Kim et al., 2006[42]; Piemonti et al., 2009[65]; Simeoni et al., 2004[80]), while high TIMP-1 serum levels were found in adolescents with alcohol intoxication (Zdanowicz et al., 2022[107]). This demonstrates the difficulty and challenges in identifying an appropriate predictive marker, given that cytokine and protein levels can be affected by various external factors, including age, diet, lifestyle, pre-existing illnesses and medication.

Notably, our study revealed that the SII levels of active smokers were significantly higher than those of non-smokers and ex-smokers. This finding is important for patients undergoing musculoskeletal surgery and suffering from infections. We demonstrated a strong correlation between smoking and an elevated systemic inflammatory response. Additionally, our findings indicate that smokers experience a prolonged inflammatory response, potentially increasing their susceptibility to inflammation-related illnesses due to their higher SII.

These higher levels were more frequently found in the group of active smokers, but not in the ex-smokers or non-smokers. The SII index, which can be calculated from platelet, neutrophil and lymphocyte counts, has been associated with various cancers, including colorectal carcinoma, small cell lung carcinoma, and cervical cancer (Hong et al., 2015[35]; Huang et al., 2019[38]; Chen et al., 2017[13]). Elevated levels of SII have also been described in postmenopausal women with osteoporosis (Zhang et al., 2023[108]; Tang et al., 2022[89]; Du et al., 2021[22]). In a study, Zhang et al. (2023[108]) detected a negative correlation between SII and bone mineral density. 

In our study, we were able to show that smokers have significantly higher SII levels overall, but that these levels do not differ when a complication occurs. This may be explained by the fact that there is already an immune response to a trauma or a chronic infection, which cannot be further increased due to exhaustion (Yang et al., 2023[104]; Wherry and Kurachi, 2015[99]). 

The study's findings confirmed that smoking has a detrimental effect on postoperative recovery and immune response. This is in line with previous research that has demonstrated an increased risk of postoperative complications, including infections, wound healing disorders and bone healing disorders (Hess et al., 2020[33]; Mills et al., 2011[53]; Pearson et al., 2016[61]; Gaston and Simpson, 2007[27]). Smoking may reduce blood flow and oxygen supply to tissues, which can lead to delayed or impaired wound and bone healing, potentially explaining the higher complication rate and longer hospital stay in smokers (Leow and Maibach, 1998[47]; Sorensen et al., 2009[86]; Gullihorn et al., 2005[30]). 

Additionally, the study reveals that smoking modifies the expression of various proteins that regulate inflammation and the immune response. One explanation for the change in expression in smokers is that smoking causes chronic systemic inflammation (Caliri et al., 2021[9]; Wang et al., 2022[94]), which leads to immune dysregulation. However, none of the proteins analyzed is really suitable as a predictive factor for complications in order to be used as a predictive blood marker. This is probably because protein levels can be affected not only by smoking but also by other factors such as pre-existing conditions, medication, or alcohol consumption. Therefore, it may be impossible to identify a single predictive factor.

When interpreting the results, some limitations should be considered. We measured a limited subset of proteins involved in inflammation and immune response *via* ELISA according to our array data. To obtain a more complete picture of immunological changes in smokers, a more comprehensive analysis of the entire cytokine and immune checkpoint protein profile would be recommendable. Secondly, we only considered short-term postoperative outcomes, which may not reflect the long-term effects of smoking on recovery and immune response. A longer follow-up of patients is necessary to assess the long-term consequences and cost-benefit analysis of smoking and its cessation.

In summary, this study demonstrates the impact of smoking on post-musculoskeletal surgery outcomes. The findings indicate that smoking is linked to a higher rate of complications, longer hospital stays, and increased SII levels. The results unequivocally demonstrate the detrimental effects of smoking on post-surgery recovery and highlight the urgent need for smoking cessation interventions. Additionally, the study reveals that smoking affects various proteins. However, none of the analyzed proteins is suitable for reliably predicting complications in smoking patients. 

## Declaration

### Data availability 

All data generated or analyzed during this study are included in this published article and are available from the corresponding author upon request.

### Acknowledgments

We would like to express our gratitude to the Federal Ministry of Economics and Climate Protection for partially funding this project (01MK2000G) in the AIQNET consortium. We are also grateful for the excellent support by the clinical research center of the BG Trauma Center Tübingen. In the same line, we would like to thank the IT department for outstanding IT support in patient data acquisition and Bianca Braun for her excellent technical assistance. Part of this work was carried out by L.P.S. for her medical dissertation. 

### Author contributions

L.T.: data analysis, figure preparation and manuscript writing. L.P.S: data analysis, review and editing. R.B. & M.M.H. data analysis, supervision, review and editing. R.H.A.W: review and editing. M.M.M: manuscript writing, review and editing. G.B.: statistical consulting and review. T.H.: review and editing. S.E.: supervision, review and editing. A.K.N.: supervision, data discussion, interpretation, critical manuscript revision and editing, project administration and funding acquisition.

### Conflict of interest

The authors declare that they have no conflict of interest.

## Figures and Tables

**Table 1:**
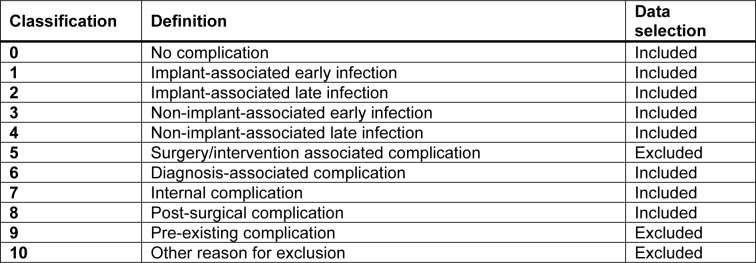
Overview of complications

**Table 2:**
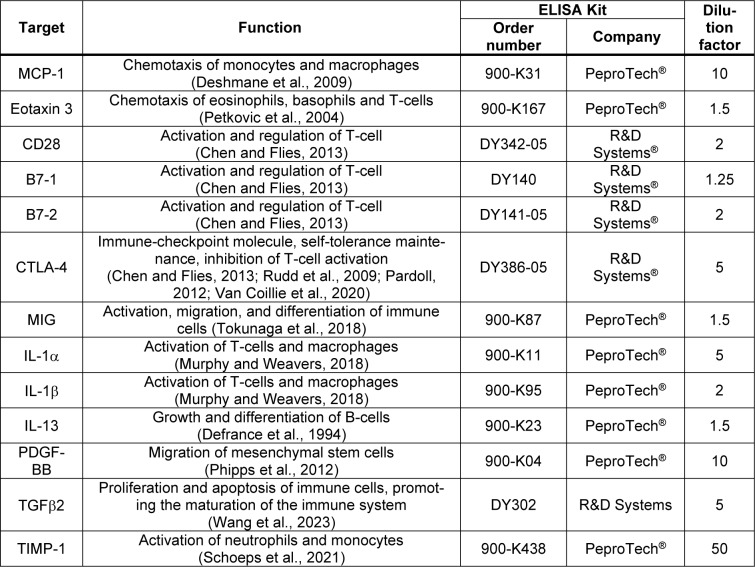
Overview of the performed enzyme-linked immunosorbent assays (ELISAs)

**Figure 1:**
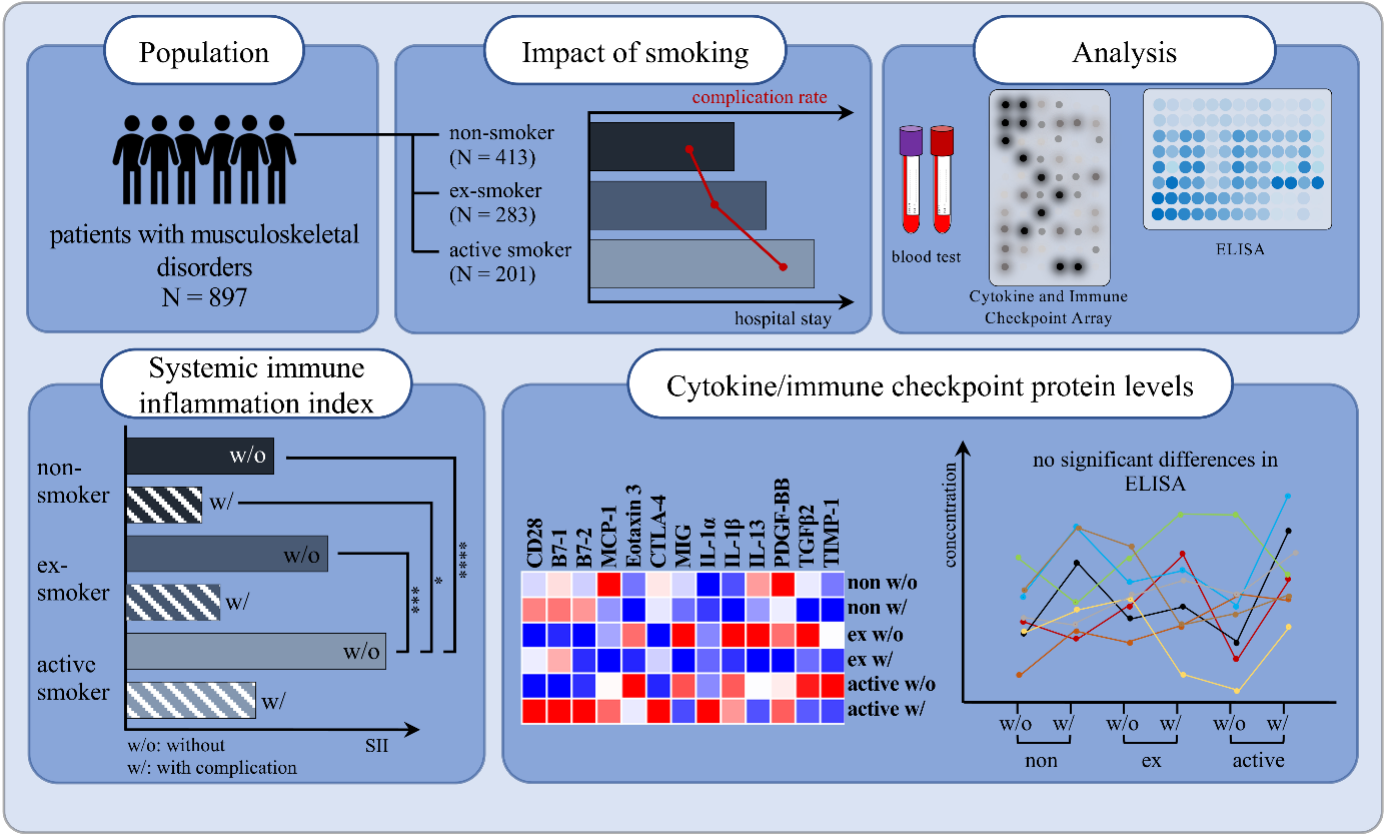
Graphical abstract
